# A widespread group of large plasmids in methanotrophic *Methanoperedens* archaea

**DOI:** 10.1038/s41467-022-34588-9

**Published:** 2022-11-18

**Authors:** Marie C. Schoelmerich, Heleen T. Ouboter, Rohan Sachdeva, Petar I. Penev, Yuki Amano, Jacob West-Roberts, Cornelia U. Welte, Jillian F. Banfield

**Affiliations:** 1 Innovative Genomics Institute, University of California, Berkeley, CA USA; 2 Department of Microbiology, Radboud University, Nijmegen, AJ Netherlands; 3 Soehngen Institute of Anaerobic Microbiology, Radboud University, Nijmegen, AJ Netherlands; 4 Sector of Decommissioning and Radioactive Wastes Management, Japan Atomic Energy Agency, Ibaraki, Japan; 5 Environmental Science, Policy and Management, University of California, Berkeley, CA USA; 6 Earth and Planetary Science, University of California, Berkeley, CA USA; 7 Lawrence Berkeley National Laboratory, Berkeley, CA USA

**Keywords:** Metagenomics, Archaeal genomics, Environmental microbiology

## Abstract

Anaerobic methanotrophic (ANME) archaea obtain energy from the breakdown of methane, yet their extrachromosomal genetic elements are little understood. Here we describe large plasmids associated with ANME archaea of the *Methanoperedens* genus in enrichment cultures and other natural anoxic environments. By manual curation we show that two of the plasmids are large (155,605 bp and 191,912 bp), circular, and may replicate bidirectionally. The plasmids occur in the same copy number as the main chromosome, and plasmid genes are actively transcribed. One of the plasmids encodes three tRNAs, ribosomal protein uL16 and elongation factor eEF2; these genes appear to be missing in the host *Methanoperedens* genome, suggesting an obligate interdependence between plasmid and host. Our work opens the way for the development of genetic vectors to shed light on the physiology and biochemistry of *Methanoperedens*, and potentially genetically edit them to enhance growth and accelerate methane oxidation rates.

## Abstract (web summary)

Anaerobic methanotrophic (ANME) archaea obtain energy from the breakdown of methane. Here, Schoelmerich et al. describe large plasmids associated with ANME archaea of the *Methanoperedens* genus in enrichment cultures and other natural anoxic environments, opening the way for development of genetic vectors for research on these poorly understood organisms.

## Introduction

Anaerobic oxidation of methane (AOM) is a microbial process of a polyphyletic group of archaea termed ANME. While most known ANME inhabit marine environments and rely on a syntrophic partner (ANME-1, ANME-2a-c, ANME-3), the *Methanoperedenaceae* (formerly ANME-2d) live in freshwater ecosystems and use nitrate, iron oxide, or manganese oxide as extracellular electron acceptors^1–4^. AOM has sparked increasing interest due to its role in naturally decreasing CH_4_ emissions by reoxidizing it to CO_2_. Methanogenic archaea (methanogens) make CH_4_ using either CO_2_, methylated compounds, or acetate as the carbon source^5^. ANME seem to reverse the methanogenesis process by using largely the same enzymatic machinery in a process termed “reverse methanogenesis”^6,7^. Understanding their metabolism and how it is regulated is of increasing interest, due to their ecological importance in the global CH_4_ cycle.

The number of known extrachromosomal elements (ECEs) in the archaeal domain of life is still limited. Most originate from a narrow range of *Sulfolobaceae*, *Haloarchaeaceae*, and *Thermococcaceae*, and some methanogens^8^. They have been primarily discovered through isolation, and this has been very important for developing genetic tools for archaea^8–11^, but their native functions are not well established. Metagenomics is a powerful method that has led to an accelerated discovery of new plasmid sequences, yet of all 38,286 plasmid sequences that are available on NCBI, only 334 originate from the archaeal domain of life (https://www.ncbi.nlm.nih.gov/genome/browse#!/plasmids/, May 24, 2022).

The recent discovery of huge ECEs associated with methane-oxidizing members of the *Methanoperedens* has ignited interest in finding ways to understand and potentially leverage these novel ECEs for genetic engineering purposes^12^. These ECEs are unlike known plasmids or viruses, yet seem to have assimilated DNA from their host and were thus coined Borgs (in analogy to Star Trek). Here, we describe the discovery of *Methanoperedens* plasmids in metagenomic datasets originating from two bioreactors as well as several natural ecosystems. We manually curated two plasmid genomes to completion. The genetic repertoire and expression profile of the plasmids is presented, and elements for a shuttle vector for future genetic engineering approaches are identified. We anticipate that this discovery will lead to important advances in understanding the ecology, physiology, biochemistry, and bioenergetics of ANME archaea.

## Results

### Search for ECEs revealed large plasmids

To find plasmids that associate with *Methanoperedens* we searched for contigs with plasmid-like gene content and taxonomic profiles most similar to those of *Methanoperedens* but that were not part of a *Methanoperedens* chromosome in metagenomic datasets from two bioreactors that are dominated by “*Candidatus* Methanoperedens BLZ2” (Bioreactor 1^13^) and “*Candidatus* Methanoperedens nitroreducens Vercelli” (Bioreactor 2^14^). The bioreactors have been maintained since 2015 and the main metabolism of both enrichment cultures is nitrate-dependent AOM. Samples for DNA and RNA extractions were taken in April 2021 and again in October 2021. “*Ca*. Methanoperedens BLZ2” comprised ~44% of the sampled community in Bioreactor 1. It has a ~3.93 Mbp genome and coexists with *Methylomirabilis oxyfera* with a ~2.73 Mbp genome that accounted for 26% of the organisms in the sampled community, whereas all other organisms were < ~5%. “*Ca*. Methanoperedens nitroreducens Vercelli” in Bioreactor 2 constituted ~78% of the sampled community. It has a ~3.28 MBp genome and coexists with many other microorganisms, each of which comprises <4% of the community.

We found two plasmids in Bioreactor 1: HMp_v1 and HMp_v5 and two plasmids in Bioreactor 2: HMp_v2 and HMp_v3 (Table 1), both of which are distinct from the plasmids in Bioreactor 1. Importantly, *Methanoperedens* are the only archaea that coexist with these archaeal plasmids and this enabled us to confidently assign “*Ca*. Methanoperedens BLZ2” (4357x coverage) as the host of HMp_v5 (4599x coverage) and “*Ca*. Methanoperedens nitroreducens Vercelli” (4204x coverage) as the host of HMp_v2 (5405x coverage). HMp_v3 (19x coverage) may be a plasmid of Mp_Bioreactor_2_Methanoperedens_40_26 (26x coverage) or a rare plasmid of the Vercelli strain. No alternative potential host was identified for HMp_v1 (27x coverage), so this may be a second rare plasmid of “*Ca*. Methanoperedens BLZ2”. Overall, we infer that the abundant plasmids are maintained at the same copy number as the *Methanoperedens* chromosome. This parallels findings for *Halobacteriales*, which usually have the same copy number of chromosomes and megaplasmids^15^.

**Table 1:** Features of *Methanoperedens* plasmids

Name	Ecosystem	Genome	Site	Size (bp)	% GC	Cov	# Ctg	# Features
**HMp_v1**	Bioreactor	complete	Bioreactor 1	191,912	39.2	27	1	186
**HMp_v2**	Bioreactor	complete	Bioreactor 2	155,605	39.2	5405	1	159
**HMp_v3**	Bioreactor	partial	Bioreactor 2	63,092	38.9	19	1	56
**HMp_v5**	Bioreactor	almost complete	Bioreactor 1	185,698	39.7	4599	1	164
**HMp_v4**	Environmental	fragment	SRVP	35,271	40.9	8	8	35
**HMp_v6**	Environmental	fragment	Japan	24,153	40.4	15	1	25
**HMp_v7**	Environmental	partial	Rifle	163,653	41.2	17	3	125
**HMp_v8**	Environmental	almost complete	SRVP	253,501	41.5	20	9	288

**Fig. 1:**
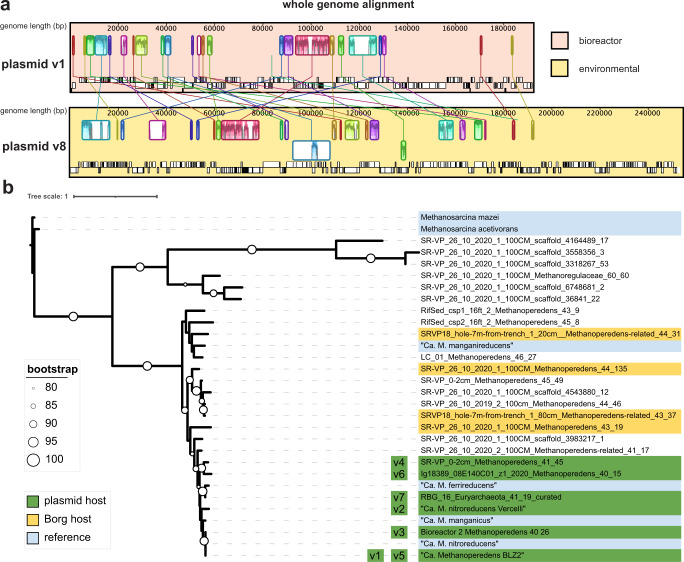
Genome alignment of plasmid versions v1 and v8 and phylogenetic tree of different *Methanoperedens* species. **a** All nine contigs of HMp_v8 were sorted and aligned onto the complete genome of HMp_v1 using the MCM algorithm in Geneious. Homologous regions of sequence shared by both plasmids (collinear blocks) are highlighted in the same color. **b** Ribosomal protein 3 (rpS3)-based phylogenetic tree of *Methanoperedens* species rooted on rpS3 of the methanogen *M. mazei*. Plasmid-host associations are inferred for plasmids 1–7 based on co-occurrence and the same coverage.

We then searched for additional sequences in our metagenomic database and identified four related plasmids from three different ecosystems (Table 1). A nucleotide alignment of all contigs from each bin to the curated plasmid versions v1 and v2 revealed homologous regions between the plasmids (Fig. 1a and Fig. S1). These sequences originated from the sedimentary rock Horonobe Japan Deep Subsurface research site^16^, a shallow aquifer adjacent to the Colorado River (Rifle, CO, USA;^17^), and saturated wetland soil (Lake County, CA, USA;^18^). Thus, we suggest that plasmids may often be associated with certain *Methanoperedens* species. The Horonobe plasmid, HMp_v6, only co-occurs with one *Methanoperedens* species (Ig18389_08E140C01_z1_2020_Methanoperedens_40_15) that is at very similar coverage to the plasmids (both 15x coverage). The Rifle plasmid HMP_v7 (17x coverage) occurs in a sample with many archaea, but we only identified one as a *Methanoperedens* species, RBG_16_Methanoperedens_41_19 (19x coverage^17^). Thus, we also suspect a plasmid-host ratio of ~1:1 for these environmental plasmids.

We constructed a phylogenetic tree to examine the pattern of associations between various *Methanoperedens* species and plasmids. The bioreactors do not contain Borgs and the *Methanoperedens* in the bioreactors are not closely related to species that host Borgs (Fig. 1b). Only in the case of HMp_v4 and HMp_v8 do Borgs and plasmids co-occur in samples with *Methanoperedens*, but these samples contain many *Methanoperedens* species. Notably, we find that the species of *Methanoperedens* that host the plasmids are phylogenetically clustered together and are distinct from the species suggested to host Borgs. Thus, this clade of *Methanoperedens* plausibly consistently hosts the plasmids. This *Methanoperedens* species group includes “*Ca*. M. nitroreducens”, “*Ca*. M. ferrireducens” and “*Ca*. M. manganicus”, so plasmids may also occur in the enrichment cultures that contain these strains.

### Curation and completion of two plasmid sequences

**Fig. 2:**
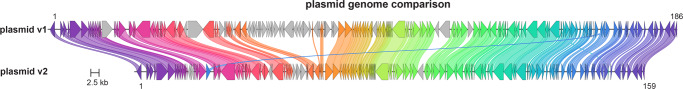
Alignment of curated HMp_v1 from Bioreactor 1 and HMp_v2 from Bioreactor 2. Colored genes highlight homologs based on amino acid identity. Gene numbers are indicated at the beginning and at the end.

Two plasmid genomes were curated to completion (see Methods). The Illumina-reads-based assembly was confirmed using long-read sequencing reads with Oxford Nanopore Technologies (Nanopore). After adjustment of the start so that it coincides with the defined start of the Illumina-based genome, the single 153,309 bp Nanopore contig supports the complete genome throughout, with the exception of occasional single Nanopore base call errors (Fig. S2). After curation, the ends of each plasmid sequence were identical and spanned by paired reads, revealing that they are circular. The plasmids carry genes on both strands and most genes are within polycistronic transcription units. HMp_v1 is 155,605 bp and has 159 ORFs, HMp_v2 is 191,912 bp and has 186 ORFs. These two plasmids do not encode tRNAs, rRNAs, or ribosomal proteins. Large stretches of v1 and v2 align, resulting in 139 shared (and mostly identical) proteins. Forty-seven proteins are unique to v1 and 19 proteins are unique to v2 (Fig. 2 and Supplementary Data 1, 5).

**Fig. 3:**
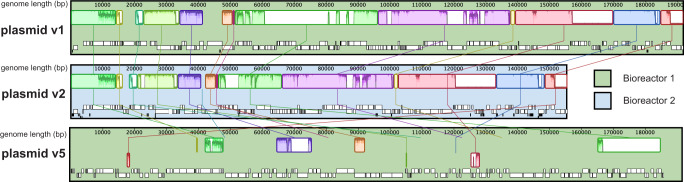
Genome alignment of curated plasmid versions v1, v2, and contig of v5. Genomes were aligned with the progressiveMauve algorithm in Geneious.

The partially curated HMp_v5 plasmid is encoded on a single 185,698 bp contig with 166 ORFs. It only aligns with HMp_v1 and HMp_v2 in some regions, indicating that it is more distantly related than v1 is to v2 (Fig. 3). It encodes ribosomal protein uL16 (Bioreactor_1_104068_82). No uL16 gene was identified in *Methanoperedens* in the bioreactor or on any unbinned contigs in the metagenome. Encoded adjacent to uL16 on HMp_v5 is translation initiation factor 2 subunit beta (aeIF-2b) that also appears to be missing from the host genome. HMp_v5 also encodes tRNA Asp, tRNA Arg, and tRNA Val. Interestingly, the host appears to lack tRNA Asp and the anticodons of the plasmid tRNA Val and tRNA Arg are not represented in the tRNA inventory of the host. The plasmid tRNAs group phylogenetically with tRNAs from other species of the same class as *Methanoperedens* (*Methanomicrobia*). Thus, the plasmid tRNAs are likely derived from *Methanomicrobia* (Fig. S3–S5).

### Plasmid replication, stability, and segregation

**Fig. 4:**
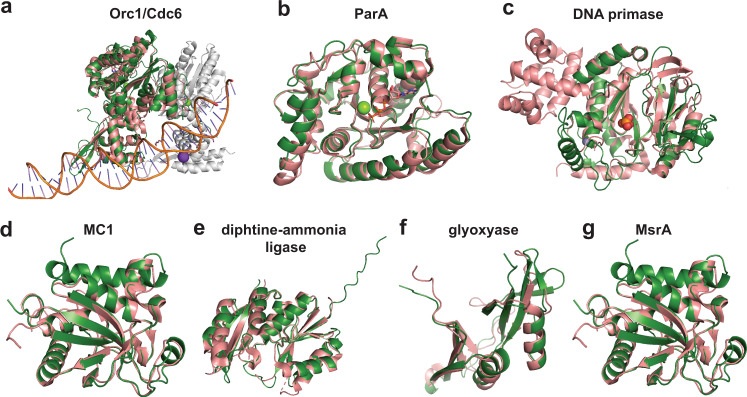
Predicted structures of plasmid proteins from HMp_v1. Plasmid proteins (green) were superimposed on best hits (salmon) from PDBeFold in pyMOL. **a** Orc1/Cdc6 (ORF1) and heterodimeric Orc1-1/Orc1-3 complex from *Sulfolobus solfataricus* (PDB: 2QBY_A, RMSD = 1.91). Orc1-3 (2QBY_B) is drawn in gray. **b** ParA (ORF167) and HpSoj from *Helicobacter pylori* (6iuc_A, RMSD = 0.9). **c** DNA primase (ORF120) and *P. furiosus* homolog (PDB: 1v33_A, RMSD = 1.91). **d** Nucleoid protein MC1 (ORF157) and DNA-archaeal MC1 protein complex from *M. thermophila* (2khl_A, RMSD = 2.30). **e** DAL (ORF67) and *Pyrococcus horikoshii* homolog (PDB: 2d13_A, RMSD = 1.37). **f** Glyoxalase (ORF36) and *Enterococcus faecalis* homolog (PDB: 2P25_A, RMSD = 1.19). **g** MsrA (ORF81) and *Mycobacterium tuberculosis* homolog (PDB: 1nwa_A, RMSD = 0.75).

We predict that the origin of replication of the plasmids is at the beginning of the sequences since it is where the origin of replication recognition complex 1 (Orc1/Cdc6) is located (Fig. 4a). Moreover, there are several repeats flanking this gene, and it is followed by a 1743 long AT-rich intergenic region. These features depict the basic structure of conserved replication origins in archaea^19^. The two complete plasmid genomes had cumulative GC skew profiles that, although weak signals, would support bidirectional replication from an origin in this region to the terminus (Fig. S6). A Cdc24-domain-bearing protein encoded elsewhere in the genome (ORF94; all ORF numbers apply to the HMp_v1 version, unless indicated otherwise) may also be involved in the progression of DNA replication. ORF2 and ORF3 fall within protein subfamilies that include sequences loosely annotated as RepA. Modeling supports their annotation as RepA1 and RepA2 with the closest structural similarity to two subunits of the trimerization core of human RepA (PDB: 1l1o:F and 1l1o:B) proteins, which are ssDNA binding proteins essential for preventing reannealing and degradation of the growing ssDNA chain during replication. The region encompassing the origin of replication and the adjacent genes encoding replication-associated proteins are likely important core elements if the plasmids are adapted into a genetic engineering vector.

The plasmids encode six helicases with variable domain topologies. ORF5 encodes a helicase with an additional N-terminal N-6 methylase domain that may unwind DNA and immediately methylate nascent DNA at the replication fork. A ubiquitin-activating (adenylating) ThiF family protein and a RadC-like protein tied to recombinational repair at the replication fork^20^ as well as a DUF488-bearing protein, nucleotide-sensing YpsA protein and UvrD helicase resembling Dna2 are also part of this genetic neighborhood. RadC (ORF11) and the UvrD helicase (ORF20) occur in many of the plasmids and were thus used to phylogenetically determine their relatedness (Fig. S7A, B). Indeed, the plasmid proteins clustered together, corroborating whole genome alignments that indicate they are related. Supplementing the sequences with top hits from BlastP search on NCBI furthermore substantiated that the plasmid proteins are most closely related to *Methanoperedens*.

Other plasmid genes are predicted to be involved in the segregation of replicated genetic material. A structure-based homology search identified ParA (Fig. 4b), but there is no obvious ParB or AspA homolog, which are the other two components of the tripartite DNA partitioning system in Crenarchaeota^21^. In both plasmids, ParA is accompanied by a transposase and a gene with an RMI2 domain that may serve the function of ParB.

The plasmids also encode an SMC chromosome segregation ATPase within a seven-gene cluster (ORFs 115-121). This protein can preside over cell-cycle checkpoints^22^. The cluster also encodes another AAA-ATPase common in archaea, together with a DNA primase that structurally aligns very well with the eukaryotic-type DNA primase of *Pyrococcus furiosus* (Fig. 4c). We infer it synthesizes an RNA primer required for the onset of DNA replication, indicating its potential importance in a vector constructed for genetic engineering. A putative DEAD/DEAH box RNA helicase (ORF34) may remodel RNA structures and RNA–protein complexes.

The plasmids encode a nucleoid protein MC1 (ORF157) that is homologous to eukaryotic histones. The predicted structure aligns well with MC1 from *Methanosarcina thermophila*, but it possesses an additional N-terminal region that is largely unstructured (Fig. 4d). We conclude that the HMp nucleoid protein MC1 is likewise responsible for plasmid genome compaction while allowing replication, repair, and gene expression.

The plasmids appear to encode multiple proteins that could be involved in DNA recombination. One is a helicase with an N-terminal UvrD helicase domain and a C-terminal PD-(D/E)XK nuclease domain (ORF40) that is also found in Cas4 nucleases. This protein was found on several HMp plasmids, as well as the genome of a large plasmid of the methanogen *Methanomethylovorans hollandica* (Fig. S7C). ORF45 and ORF128 encode HNH-endonucleases that could stimulate recombination. ORF128 has an RRXRR motif, an architecture common to some CRISPR-associated nucleases (COG3513)^23^. Furthermore, the plasmids encode a recombination limiting protein RmuC (ORF176).

The plasmids encode other genes involved in nucleotide processing. This set includes a 5-gene cluster encompassing a putative AAA-ATPase (COG1483, ORF149), two genes of unknown function, a nuclease (ORF144), and a helicase with a similar architecture to the RNA polymerase (RNAP) associated protein RapA. RapA reactivates stalled RNAP through an ATP-driven back-translocation mechanism, thus stimulating RNA synthesis^24^. Furthermore, HMp_v1 encodes a large (1550 AA) protein with an N-terminal ATPase domain and a C-terminal HNH endonuclease domain (ORF39). Interestingly, this latter domain is preceded by a 330 bp region that encodes 11 repeated [PPEDKPPEGK] amino acid sequences that are predicted to introduce intrinsic disorder. The C-terminal region resembles the histone H1-like DNA binding protein and inner and outer membrane linking protein TonB. We speculate that the repeat region facilitates the binding of the ATPase/endonuclease to other interaction partners (nucleic acid or protein).

### Transporters and membrane proteins

HMp_v2 encodes 15 membrane proteins and three extracellular proteins, whereas the larger HMp_v1 carries 25 predicted membrane proteins and four extracellular proteins. This difference is due to one large genetic island in HMp_v1 that encodes several transporters. There is a single gene encoding a Fe^2+^/Mn^2+^ transporter (ORF38) that is also found in some Asgard archaea and bacteria and a region spanning ORFs 54-79 that encodes several transport systems. First, a putative CbiMNQO Co^2+^/Ni^2+^ transporter composed of three membrane subunits and a soluble subunit whose expression could be controlled by the preceding NikR regulator. Second, an amino acid permease (ORF61) whose expression could be regulated by an accompanying HrcA. Third, a second CbiMNQO Co^2+^/Ni^2+^ transporter with similar architecture, whose expression may be controlled by an Ars regulator. Another NikR (ORF67) follows, and several proteins with the same DUF3344 are predicted to be located extracellularly and are likely cell surface proteins (ORF68, ORF70). The genetic region is completed by another ABC-transporter that could be a biotin transporter since two subunits resemble EcfT and EcfA1/2 (ORFs 72-75). This region appears not to be present in the coexisting *Methanoperedens*, indicating the potential for the plasmids to augment their host’s metabolism.

Two gene clusters flanked by transposases encode two putative membrane complexes. The first includes a secretion ATPase VirB11 and a 7-TMH-bearing membrane protein (Fig. S8). The combination is reminiscent of a system for DNA transfer between *Sulfolobus* cells^25^. The second includes multiple membrane proteins with features suggestive of binding DNA/RNA/proteins and/or lipoproteins and a HerA helicase (ORF112) of unknown localization that possesses a domain found in conjugative transfer proteins. This second cluster could be involved in the extrusion of DNA.

The HMp_v1 plasmid encodes four tetratricopeptide repeat proteins (TPR). One is a membrane protein and two are membrane-attached and cytoplasmically-orientated TPRs. The fourth is a soluble protein that is accompanied by a small 3-TMH-bearing membrane protein and is possibly tied to membrane processes in the host. The TPR domains facilitate protein-protein interactions and are, for example, required for PilQ assembly of the type IV pilus. TPR4 may be involved in the homologous archaeosortase system that cleaves the signal peptide and replaces it with another modification. The HMp_v2 plasmid carries two presumably protein-binding pentapeptide repeat-containing proteins (v2 ORFs 16-17).

### Proteins involved in cell protection

Both plasmids encode a dCTP deaminase (ORF156), which preserves chromosomal integrity by reducing the cellular dCTP/dUTP ratio, preventing the incorporation of dUTP into DNA. HMp_v1 also encodes a diphtine-ammonia ligase (DAL) (Fig. 4E) that catalyzes the last step of a post-translational modification of the elongation factor eEF2 during ribosomal protein synthesis^26^. A glyoxalase (Fig. 4F) can convert cytotoxic α-keto aldehydes into nontoxic α-hydroxycarboxylic acids^27^. ArsR may regulate the expression of a peptide methionine sulfoxide reductase (MsrA, Fig. 4G). MsrA repairs oxidative damage to methionine residues arising from reactive oxygen species and reactive nitrogen intermediates^28^.

### Expression of plasmid genes

We used metatranscriptomics to determine which of the genes of the high abundance plasmids v2, v5, and the lower abundance v1 are most important to the *Methanoperedens* growing in the bioreactors. Metatranscriptome reads from Bioreactor 1 or 2 were stringently mapped onto all contigs of each respective bioreactor. Read counts were normalized to the gene length and genes were considered expressed that had at least 0.5 mapped reads. We found reads that mapped uniquely onto all three plasmid genomes, and the high-coverage plasmids had higher normalized read counts (Supplementary Data 2).

Twenty of the 178 genes of the low-coverage plasmid HMp_v1 were expressed. Most highly expressed were the gene encoding the MTH865 protein, which has been structurally characterized but lacks a known function^29^, and its accompanying genes (ORFs 30-31). Also highly expressed were the first Co^2+^/Ni^2+^ transporter and two preceding genes. One component of the putative biotin transporter was also expressed and the nucleoid protein MC1. This suggests that this plasmid facilitates or enhances the uptake of Co^2+^/Ni^2+^ and possibly biotin.

Of the 159 genes of the high-coverage plasmid HMp_v2 in Bioreactor 2, 103 were expressed. The highest expression of genes (≥100 normalized reads) with functional annotations was observed for ParA and its genetic context, the dCTP deaminase, and MTH865. Moderate expression (≥10) was observed for genes encoding MsrA and its regulator, as well as the glyoxalase. Thus, we infer that this plasmid is actively conferring protection from oxidative stress and cytotoxic compounds. Genes that only showed low expression are mostly important for plasmid maintenance. Interestingly, the origin of replication proteins of all plasmids were not expressed. However, we detected expression of the OriC adjacent gene, encoding a hypothetical protein which has a P-loop fold. This suggests that this could be an important component in plasmid replication, possibly performing ATP hydrolysis (ORF186).

Of the 164 genes of HMp_v5 from Bioreactor 1, 104 were expressed. The most highly expressed genes of HMp_v5 are the first gene encoding a hypothetical small protein and the last gene encoding the small subunit GroES (Chaperonin Cpn10) of a three-gene cluster that includes GroEL. This chaperonin system is crucial for accurate protein folding^30^. Other highly expressed genes (≥500 normalized reads) encode a HrcA regulator, which could enable expression of the equally expressed, adjacently encoded 50 S ribosomal protein uL16 and translation initiation factor 2 subunit beta, as well as a rubrerythrin. Furthermore, another ArsR regulator, a putative integrase and a two-component system with a resemblance to FleQ, a transcriptional activator involved in the regulation of flagellar motility, were highly expressed.

Moderately expressed (≥50 normalized reads) were proteins involved in toxin-antitoxin systems, an archaeal translation initiation factor, two adjacently encoded TIR-like nucleotide-binding proteins located next to the protein involved in replication initiation and proteins involved in cell growth and apoptosis (IMPDH ParBc_2). Overall, the main function of HMp_v5 may be to ensure protein maturation and regulate DNA processes, including transcription and translation.

### Plasmid specificity of proteins

**Fig. 5:**
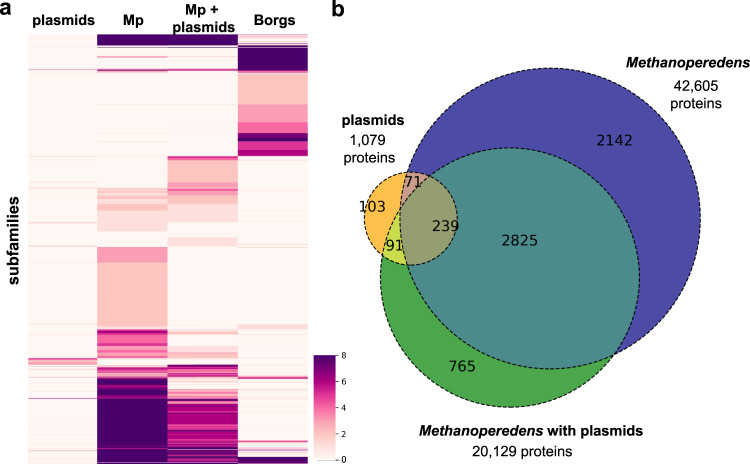
Protein clustering analyses and distribution of protein subfamilies across main elements. **a** Heatmap showing protein subfamilies (subfamilies ≥8 are all shown in dark purple). **b** Subfamily distribution across plasmids, *Methanoperedens* with plasmids and *Methanoperedens* without plasmids.

To further understand how the plasmid inventories may augment or overlap with those of the host *Methanoperedens* we performed protein family clustering using a protein dataset composed of 96,548 proteins from the HMp plasmids and *Methanoperedens* chromosomes (Supplementary Data 3). Also included were proteins from Borgs and a small set of reference proteins from plasmids of methanogens. The hierarchical clustering revealed that the plasmid proteomes clustered distinctly from *Methanoperedens* and Borg proteomes (Fig. 5a and Fig. S9). Of the 1,079 plasmid proteins, 882 (82%) clustered into 504 subfamilies. The majority of plasmid-encoded proteins had homologs in the *Methanoperedens* genomes (80%). The number of protein subfamilies exclusively shared between plasmids and their host *Methanoperedens* was slightly higher (18%) than for *Methanoperedens* without plasmids (14%) (Fig. 5B and Fig. S9).

Eighteen percent of the plasmid proteins clustered into subfamilies that were unique to the plasmids, and forty-one percent were in subfamilies with only a few non-HMp homologs (Table 2 and Supplementary Data 3). Many plasmid-enriched proteins are implicated in DNA replication and repair, including the Cdc24 protein and the DNA primase, and they were actively expressed in HMp_v2. A surprising finding was that there are no homologs on NCBI for a large surface protein that is only found on four HMp versions.

**Table 2:** Protein subfamilies enriched in plasmid proteins. Numbers in brackets indicate the number of subfamily members per plasmid

subfamily	PFAM annotation	description	# plasmid members	# total members	% enr.	plasmids	v1 ORF
subfam0052	Exo_endo_phos	nuclease	3	3	100	v1, v2, v5	144
subfam0053	DUF87 AAA_10	HerA helicase	3	3	100	v1, v2, v8	112
subfam0085	FYDLN_acid		2	2	100	v1, v2, v8	177
subfam2357	AAA_33		2	2	100	v1, v2	186
subfam2361	Cas_Cas02710	CRISPR-associated protein	2	2	100	v5, v8	
subfam2619	AAA_26	ParA2	3	3	100	v1, v2, v8	102
subfam3152	Big_3_3	surface protein	4	4	100	v1, v2, v4, v8	25
subfam3198	YAcAr		2	2	100	v1, v2	17
subfam3232	TIR-like		2	2	100	v5 (2)	
subfam4360	DUF488		3	3	100	v1, v2, v8	19
subfam4949	Pilin_N		2	2	100	v8 (2)	
subfam4974	DUF2250		3	3	100	v1, v2, v8	118
subfam5544	DUF2225 FYDLN_acid		2	2	100	v1, v2	135
subfam6115	UTP25		3	3	100	v4, v5, v8	
subfam6300	CDC24_OB3	Cdc24	3	3	100	v1, v2, v8	94
subfam7697	LexA_DNA_bind	LexR	3	3	100	v1, v2, v8	22
subfam7758	KdpD DUF2478	secretion ATPase VirB	4	4	100	v1, v2, v4, v8	89
subfam0185	RuvC_1	transposase	3	13	23	v1 (2), v3 (5), v5	43, 88
subfam0187	Piwi		3	7	43	v1, v2, v3	184
subfam0188	DNA_primase_S	DNA primase	3	4	75	v1, v2, v8	120
subfam0434	DUF1156 MethyltransfD12 N6_N4_Mtase		4	7	57	v1, v2, v4, v7	146
subfam0673	HDA2-3		5	16	31	v1, v2, v4, v5, v7	145
subfam1345	HTH_29		2	3	66	v1, v2	130
subfam1876	CCDC-167		3	14	21	v1, v2, v8	96
subfam1990	ThiF	ThiF	6	8	75	v1, v2, v3, v5, v8 (2)	7
subfam2654	UPF0259		3	12	25	v1, v2, v5	183
subfam3527	AAA_11 PhoH		7	22	32	v1, v2, v5 (2), v6, v7 (2)	40
subfam4932	AAA_11 AAA_12	UvrD helicase	5	7	71	v1 (2), v2, v3, v8	20, 123
subfam5002	RMI2	RepA2	8	65	12	v1 (2), v2 (2), v3, v5, v7, v8	3, 169
subfam6245	YpsA	YpsA	5	7	71	v1, v2, v5, v7, v8	18
subfam6302	DUF3584 Macoilin Cast MAD MAD AAA_13	chromosome segregation ATPase	3	6	50	v1, v2, v8	117
subfam6760	NARP1 SPO22	TPR3	3	9	33	v1, v2, v3	178
subfam6769	CDC24_OB3	RepA1	6	58	10	v1, v2, v3, v5, v7, v8	2
subfam7198	UTP25 DUF1998	RNA helicase	4	6	67	v1, v2, v4, v5	34
subfam7280	Integrin_beta	Integrin	4	6	67	v1, v2, v3, v8	129
subfam7300	RRXRR RE_Alw26IDE	HNH endonuclease	6	31	19	v1, v7, v8 (4)	128
subfam7324	RadC	RadC	5	41	12	v1, v3, v5, v7, v8	11
subfam7387	RuvC_1		2	3	67	v4, v8	
subfam7760	ChAPs	TPR1	4	5	80	v1, v2, v5, v6	28

There were a few instances of plasmid proteins that are auxiliary/linked to central metabolic functions, for example, a protein responsible for removing ammonia from glutamine (HMp_v5), a putative cobalamin-independent methionine synthase implicated in amino acid metabolism (HMp_v7), and a multiheme cytochrome (MHC) that may be important for electron transfer to the final electron acceptor of CH_4_ oxidation (HMp_v7). Other proteins shared with *Methanoperedens* are potentially involved in sensing and signaling. For example, a TPR protein (HMp_v1, v2, v8), a putative nitroreductase which could function in FMN storage (HMp_v8), a phosphoglucomutase/phosphomannomutase possibly tied to glycosylation (HMp_v5), a methyltransferase involved in RNA capping in eukaryotes (HMp_v2, v8), an rRNA methylase (HMp_v5) that could be implicated in post-transcriptional modification, a translation initiation factor (HMp_v5)^31^, a peptidyl-tRNA hydrolase involved in releasing tRNAs during translation (HMp_v7) and a phosphoribosyltransferase implicated in stress response (HMp_v7)^32^.

HMp_v8 carries two IS200-like transposases and a homolog is also found on the *Methanosarcina barkeri* 227 plasmid (WP_048116267.1). There are two subfamilies that are phage integrases, one of which is also found on *Methanococcus maripaludis* C5 plasmid pMMC501 (WP_010890222.1) and *Methanosarcina acetivorans* plasmid pC2A (WP_010891114.1).

## Discussion

In two laboratory-scale bioreactors and three different natural ecosystems, we discovered large, circular plasmids of *Methanoperedens*. To our knowledge, these are the first reported plasmid sequences in the archaeal family *Candidatus* Methanoperedenaceae and the first in ANME archaea. Notably, the deduced hosts for the plasmids are a distinct *Methanoperedens* species group that includes all strains that are currently in laboratory cultures (to our knowledge). For example, “*Ca*. Methanoperedens BLZ2” (Bioreactor 1) carries HMp_v5 and “*Ca*. M. nitroreducens Vercelli” (Bioreactor 2) carries HMp_v2. The plasmids are large compared to most plasmids of methanogens (4,440–58,407 bp). The only exception is the report of a 285,109 bp plasmid of the obligately methylotrophic methanogen *M. hollandica* DSM 15978^33^.

Although our data indicate that some *Methanoperedens* may carry more than one plasmid, the most abundant (main) plasmid appears to be maintained at an ~1:1 ratio with the host chromosome. Thus, there seems to be coordination of replication of the plasmid and the main chromosome, as has been observed for other archaeal chromosomes and their megaplasmids^15^. Maintaining a large plasmid at the same abundance as the main chromosome comes at an energetic cost, suggesting that the plasmids confer cellular fitness or are possibly even essential for the host’s survival.

Interestingly, we identified Orc1/Cdc6 near the origin of replication, but these genes were not expressed at the time of sampling. This could simply be due to the very slow growth rate of *Methanoperedens* in the bioreactors, and the concomitantly low replication rates of its plasmids. Since the plasmids do not encode recombinase RadA, this excludes the possibility of origin-less replication initiation via homologous recombination as described for some viruses and archaea^34^. It would, however, also be possible that the host Orc1/Cdc6 is used to couple replication of the plasmid to chromosome copy number.

There are different versions of the plasmids, but they share elements of core machinery likely essential for plasmid replication and maintaining DNA integrity. There are also unique functions for different plasmid versions. The observation that HMp_v1 encodes highly expressed genes for Ni^2+^/Co^2+^ transporters is interesting because Ni^2+^ is required for Mcr, the enzyme complex central to methane oxidation, and for the carbon monoxide dehydrogenase/acetyl-CoA synthase. Co^2+^ is part of a complex organometallic cofactor B12, which is essential for the function of methyltransferases^35^. HMp_v2 lacks the genomic island rich in transporters and the metatranscriptomic data indicates that one of its functions is to protect the host from oxidative stress and cytotoxic compounds. HMp_v5, on the other hand, predominantly expressed genes tied to protein maturation and regulation of cellular functions, often connected to nucleotide mechanisms. Interestingly, HMp_v5, but not its host’s chromosome, carries the 50 S ribosomal protein uL16 and an adjacent gene encoding translation initiation factor 2 subunit β, essential genes for the construction of functional ribosomes and translation^36,37^. This suggests that *Methanoperedens* is dependent on the HMp_v5 plasmid, ensuring plasmid retention. The relocation of uL16 to an extrachromosomal element is reminiscent of the relationship in eukaryotes between mitochondrial DNA and nuclear DNA, where many mitoribosomal proteins are encoded in the nuclear DNA. In the case of the plasmid, this control of uL16 could ensure that increased host ribosome production leads to increased translation of plasmid genes.

Based on the phylogenetic analysis, we inferred that plasmids occur in *Methanoperedens* species that do not host Borgs. It was suggested that Borgs are not obviously plasmids, but a limitation on their classification was the lack of archaeal plasmids generally, and *Methanoperedens* plasmids, specifically, to compare them to. Here, we find that, in contrast to Borgs, the plasmids do not, or only very rarely (e.g., one MHC), carry genes with a protein function associated with the central metabolism of their host (anaerobic methane oxidation). The observations presented here underline the distinction between Borg extrachromosomal elements and plasmids of *Methanoperedens*.

Although archaeal methanotrophs of the genus *Methanoperedens* have been studied using cultivation-independent^38^ and enrichment-based methods^3^, many questions regarding their physiology remain. We hope that this discovery of naturally occurring plasmids associated with *Methanoperedens* in stable enrichment cultures, paired with the possibility of editing the genomes of specific organisms in microbial communities^39^, is a first step towards developing genetic modification approaches to better understand anaerobic oxidation of methane and potentially to harness this process for agricultural and climate engineering.

## Methods

### Identification of ECEs associated with *Methanoperedens* and manual genome curation

Metagenomic datasets on ggKbase (ggkbase.berkeley.edu) were searched for contigs with a dominant taxonomic profile matching *Methanoperedens* (Archaea; Euryarchaeaota; Methanomicrobia; Methanosarcinales; Candidatus Methanoperedens; Candidatus Methanoperedens nitroreducens). Manual genome binning was performed based on coverage, GC content, and contig taxonomy. Plasmids were identified based on marker proteins (Orc1/Cdc6) and whole genome alignments using the progressiveMauve algorithm. Additional plasmids in environmental metagenome datasets were identified by BLAST and verified by genome alignment to a bioreactor plasmid^40^. Manual curation of two plasmid sequences to completion was performed in Geneious Prime 2021.2.2 (https://www.geneious.com). Curation involved piecing together and extending contigs with approximately the same GC content, depth of sampling (coverage), and phylogenetic profile. Sequence accuracy and local assembly error correction made use of read information, following methods detailed in ref. 41. The final, extended sequences contained identical regions at the termini, and were thus circularized. The start positions of the genomes were chosen based on cumulative GC skew information.

### Nucleic acid extractions from the *Methanoperedens* enrichment cultures

DNA and RNA samples were taken from Bioreactor 1 in April 2021. DNA samples were taken from Bioreactor 2 in April 2021 and RNA samples were taken from a subculture of Bioreactor 2 in October 2021. DNA was isolated following the Powersoil DNeasy kit protocol, with the addition of a 10 min bead beating step at 50 s^−1^ (Qiagen, Hilden, Germany). RNA was isolated following the Ribopure-Bacteria kit protocol (Thermo Fisher Scientific, Waltham, US), with the addition of a step homogenizing the cells and a 15 min bead beating step at 50 s^−1^. The metatranscriptomic datasets were constructed from technical replicates (*n* = 4 for Bioreactor 1, *n* = 3 for Bioreactor 2). Plasmids were targeted in a second bioreactor sampling experiment (*n* = 2 for both bioreactors in October 2021) for which the Plasmid Miniprep kit was used according to the manufacturer’s instruction (Thermo Fisher Scientific, Waltham, US), with the addition of a step homogenizing the cells before processing. In August 2022, DNA was extracted from the bioreactors again as in October 2021 to be used for long-read sequencing. The metagenomic datasets were constructed from biological replicates (*n* = 2–4).

### Metagenomic and metatranscriptomic dataset generation

DNA was submitted for Illumina sequencing at Macrogen or at the in-house facility of Radboud University to generate 150 or 250 bp paired-end (PE) reads for metagenomes, and 100 bp PE for metatranscriptomes. Sequencing adapters, PhiX, and other Illumina trace contaminants were removed with BBTools (v38.79) and sequence trimming was performed with Sickle (v1.33). The filtered reads were assembled with IDBA-UD^42^ (v1.1.3) or MEGAHIT^43^ (v1.2.9), ORFs were predicted with Prodigal^44^ (v2.6.3) and functionally annotated by comparison to KEGG, UniRef100, and UniProt using USEARCH^45^ (v10.0.240).

The metatranscriptomic reads of Bioreactor 1 or 2 and replicate 1 or 2 were mapped against all contigs from the same sample using BBMap and a stringent mapping where reads had to be 99% identical to map (minid=0.99 ambiguous=random)^46^. The mapped reads per gene were calculated with featureCounts (--fracOverlapFeature 0.1)^47^ (v2.0.3). The resulting read counts were normalized to gene length and are given as the number of reads per 1,000 bp. Normalized reads ≥0.5 were considered expressed.

The percentage of reads that mapped onto the plasmid sequences from the plasmid isolation dataset was calculated using SeqKit^48^ (v0.12.0) and SAMtools^49^ (v1.12). Out of all 2,235,458 total reads from Bioreactor 1 replicate 1 (3,660,768 replicate 2), 0.2% (0.3%), and 1.3% (1.6%) of reads mapped on HMp_v1 and HMp_v5, respectively. Out of all 3,278,364 total reads from Bioreactor 2 replicate 1 (3,897,908 replicate 2), 4.5% (3.8%) of reads mapped on HMp_v2.

Long-read sequencing was performed using the MinION Mk1C device at the in-house facility of Radboud University. The libraries were prepared using plasmid DNA extracted from each bioreactor (1024 ng from Bioreactor 1 and 455 ng from Bioreactor 2) and the Ligation Sequencing Kit 1D (SQK-LSK109) in combination with the Native Barcoding Expansion Kit (EXP-NBD104). FastQ files were generated and demultiplexed using Guppy basecaller (v.6.1.5) in the fast basecalling setting. Adapters were trimmed with porechop^50^(v.0.2.4) and long-reads were assembled with flye^51^ (v.2.9-b1768) in meta mode.

### Nucleotide alignments and phylogenetic tree construction

Whole genome alignments were done in Geneious using the progressiveMauve algorithm when aligning complete genomes or single contigs, or the MCM algorithm when aligning genomes on multiple contigs. RpS3, UvrD, RadC and helicase/nuclease genes were aligned with MAFFT^52^ (v7.453), trimmed with trimal (-gt 0.2)^53^ (v1.4.rev15) and a maximum-likelihood tree was calculated in IQ-Tree (-m TEST -st AA -bb 1000 -nt AUTO -ntmax 20 -pre)^54^. The trees were visualized and decorated in iTOL^55^. tRNA alignments were constructed by adding predicted host and phage tRNAs to archaeal tRNA alignments from GtRNAdb release 19^56^ with the add option of MAFFT^52^ (v7.453). Using these alignments, tRNA phylogenies were constructed with IQ-tree, using the automatic model finder and 1000 bootstrap replications^54^. The trees were visualized and decorated in iTOL^55^.

### Structural, functional, and localization predictions

Proteins were profiled using InterProScan^57^ (v5.51-85.0) and HMMER (hmmer.org) (v3.3, hmmsearch) against the PFAM (--cut_nc) and KOFAM (--cut_nc) HMM databases^58,59^. TMHs were predicted with TMHMM^60^ (v2.0) and cellular localization using PSORT^61^ (v2.0, archaeal mode). tRNAs were searched with tRNAscan^56^ (v.2.0.9) and rRNAs with SSU-ALIGN^62^ (v0.1.1). Plasmid protein structures were modeled using AlphaFold2^63^ via a LocalColabFold^64^ (--use_ptm --use_turbo --num_relax Top5 --max_recycle 3), visualized and superimposed onto PDB structures using PyMOL^65^ (v2.3.4). Structure-based homology search was performed in PDBeFold^66^. The plasmid comparison figure was generated with clinker^67^ (v0.0.21).

### Protein family clustering

A dataset of 96,548 proteins was constructed using the elements in the project folders listed in the Data availability statement. These cover all eight HMp versions, four complete Borg genomes, additional incomplete Borg genomes, and *Methanoperedens* genomes. This core dataset was supplemented with reference genomes comprising protein sequences from plasmids of methanogens, and from “*Ca*. Methanoperedens nitroreducens”, “*Ca*. M. ferrireducens”, “*Ca*. M. manganicus” and “*Ca*. M. manganireducens” (Supplementary Data 4). All proteins were clustered into protein subfamilies using MMseqs2^68^ and an all-vs.-all search (e-value: 0.001, sensitivity: 7.5, and cover: 0.5). A sequence similarity network was built based on pairwise similarities and the greed set cover algorithm from Mmseqs2 was performed to define protein subfamilies. HMMs were constructed for these subfamilies based on the results2msa parameter of MMseqs2 using HHblits^69^. They were then profiled against the PFAM database by HMM-HMM comparison using HHsearch^70^. Protein subfamilies enriched in plasmid proteins were then determined by calculating the Fisher exact statistic in scipy.stats (altnerative = “two-sided”) and a subsequent false-discovery rate (FDR) correction using the *multipletests* function in statsmodels.stats.multitest (method = “fdr_bh”). Subfamilies were considered enriched with ratios ≥1 and an FDR-corrected *p* value ≤0.05. This resulted in 125 protein subfamilies that were enriched in plasmid genomes.

### Replication prediction by GC skew analysis

Replichores were predicted by calculating the GC skew (G-C/G + C) and cumulative GC skew using the iRep package (gc_skew.py)^71^.

### Reporting summary

Further information on research design is available in the Nature Portfolio Reporting Summary linked to this article.

## Supplementary information

Supplementary Information

Description of Additional Supplementary Files

Supplementary Data 1–5

Supplementary Data 6

Reporting Summary

## Data Availability

Metagenomics and metatranscriptomics sequencing reads, newly released plasmid genomes, and “*Candidatus* Methanoperedens spp.” metagenomes reported in this paper are available under NCBI BioProject: PRJNA850006. Reference datasets comprising additional “*Candidatus* Methanoperedens spp.” and Borg genomes are available under NCBI BioProject: PRJNA866293. The HMp_v2 contig assembled from Nanopore long-reads is provided in Supplementary Data 6. Sequence databases used include KEGG, UniRef100, UniProt, pfam, and ggkbase (ggkbase.berkeley.edu).

## References

[1] Haroon MF (2013). Anaerobic oxidation of methane coupled to nitrate reduction in a novel archaeal lineage. Nature.

[2] Ettwig KF (2016). Archaea catalyze iron-dependent anaerobic oxidation of methane. Proc. Natl Acad. Sci. USA.

[3] Leu AO (2020). Anaerobic methane oxidation coupled to manganese reduction by members of the Methanoperedenaceae. ISME J..

[4] Cai C (2018). A methanotrophic archaeon couples anaerobic oxidation of methane to Fe(III) reduction. ISME J..

[5] Thauer RK (2019). Methyl (Alkyl)-coenzyme M reductases: nickel F-430-containing enzymes involved in anaerobic methane formation and in anaerobic oxidation of methane or of short chain alkanes. Biochemistry.

[6] Krüger M (2003). A conspicuous nickel protein in microbial mats that oxidize methane anaerobically. Nature.

[7] Hallam SJ (2004). Reverse methanogenesis: testing the hypothesis with environmental genomics. Science.

[8] Wang H, Peng N, Shah SA, Huang L, She Q (2015). Archaeal extrachromosomal genetic elements. Microbiol. Mol. Biol. Rev..

[9] Wu Z, Liu H, Liu J, Liu X, Xiang H (2012). Diversity and evolution of multiple orc/cdc6-adjacent replication origins in haloarchaea. BMC Genomics.

[10] Bokranz M, Klein A, Meile L (1990). Complete nucleotide sequence of plasmid pME2001 of Methanobacterium thermoautotrophicum (Marburg). Nucleic Acids Res..

[11] Metcalf WW, Zhang JK, Apolinario E, Sowers KR, Wolfe RS (1997). A genetic system for Archaea of the genus Methanosarcina: liposome-mediated transformation and construction of shuttle vectors. Proc. Natl Acad. Sci. USA.

[12] Al-Shayeb, B. et al. Borgs are giant genetic elements with potential to expand metabolic capacity. *Nature*10.1038/s41586-022-05256-1 (2022). 10.1038/s41586-022-05256-1 PMC9605863 36261517

[13] Arshad A (2015). A metagenomics-based metabolic model of nitrate-dependent anaerobic oxidation of methane by methanoperedens-like archaea. Front. Microbiol..

[14] Vaksmaa A (2017). Enrichment of anaerobic nitrate-dependent methanotrophic “Candidatus Methanoperedens nitroreducens” archaea from an Italian paddy field soil. Appl. Microbiol. Biotechnol..

[15] Breuert S, Allers T, Spohn G, Soppa J (2006). Regulated polyploidy in halophilic archaea. PLoS ONE.

[16] Hernsdorf AW (2017). Potential for microbial H2 and metal transformations associated with novel bacteria and archaea in deep terrestrial subsurface sediments. ISME J..

[17] Anantharaman K (2016). Thousands of microbial genomes shed light on interconnected biogeochemical processes in an aquifer system. Nat. Commun..

[18] Crits-Christoph A, Diamond S, Al-Shayeb B, Valentin-Alvarado L, Banfield JF (2014). A widely distributed genus of soil Acidobacteria genomically enriched in biosynthetic gene clusters. ISME Commun.

[19] Wu Z, Liu J, Yang H, Xiang H (2014). DNA replication origins in archaea. Front. Microbiol..

[20] Saveson CJ, Lovett ST (1999). Tandem repeat recombination induced by replication fork defects in Escherichia coli requires a novel factor, RadC. Genetics.

[21] Schumacher MA (2015). Structures of archaeal DNA segregation machinery reveal bacterial and eukaryotic linkages. Science.

[22] Long SW, Faguy DM (2004). Anucleate and titan cell phenotypes caused by insertional inactivation of the structural maintenance of chromosomes (smc) gene in the archaeon Methanococcus voltae. Mol. Microbiol..

[23] Majorek KA (2014). The RNase H-like superfamily: new members, comparative structural analysis and evolutionary classification. Nucleic Acids Res..

[24] Liu B, Zuo Y, Steitz TA (2015). Structural basis for transcription reactivation by RapA. Proc. Natl Acad. Sci. USA.

[25] van Wolferen M, Wagner A, van der Does C, Albers S-V (2016). The archaeal Ced system imports DNA. Proc. Natl Acad. Sci. USA.

[26] Zhang Y (2010). Diphthamide biosynthesis requires an organic radical generated by an iron-sulphur enzyme. Nature.

[27] He MM, Clugston SL, Honek JF, Matthews BW (2000). Determination of the structure of Escherichia coli glyoxalase I suggests a structural basis for differential metal activation. Biochemistry.

[28] Taylor AB, Benglis DM, Dhandayuthapani S, Hart PJ (2003). Structure of *Mycobacterium tuberculosis* methionine sulfoxide reductase A in complex with protein-bound methionine. J. Bacteriol..

[29] Lee GM, Edwards AM, Arrowsmith CH, McIntosh LP (2001). NMR-based structure of the conserved protein MTH865 from the archaeon Methanobacterium thermoautotrophicum. J. Biomol. NMR.

[30] Figueiredo L (2004). Functional characterization of an archaeal GroEL/GroES chaperonin system: significance of substrate encapsulation. J. Biol. Chem..

[31] Pedullà N (2005). The archaeal eIF2 homologue: functional properties of an ancient translation initiation factor. Nucleic Acids Res..

[32] Anantharaman V, Iyer LM, Aravind L (2012). Ter-dependent stress response systems: novel pathways related to metal sensing, production of a nucleoside-like metabolite, and DNA-processing. Mol. Biosyst..

[33] Lomans BP (1999). Isolation and characterization of Methanomethylovorans hollandica gen. nov., sp. nov., isolated from freshwater sediment, a methylotrophic methanogen able to grow on dimethyl sulfide and methanethiol. Appl. Environ. Microbiol..

[34] Hawkins M, Malla S, Blythe MJ, Nieduszynski CA, Allers T (2013). Accelerated growth in the absence of DNA replication origins. Nature.

[35] Zhang Y, Rodionov DA, Gelfand MS, Gladyshev VN (2009). Comparative genomic analyses of nickel, cobalt and vitamin B12 utilization. BMC Genomics.

[36] Nierhaus KH (1991). The assembly of prokaryotic ribosomes. Biochimie.

[37] Maone E (2007). Functional analysis of the translation factor aIF2/5B in the thermophilic archaeon Sulfolobus solfataricus. Mol. Microbiol..

[38] Ino K (2018). Ecological and genomic profiling of anaerobic methane-oxidizing archaea in a deep granitic environment. ISME J..

[39] Rubin BE (2022). Species- and site-specific genome editing in complex bacterial communities. Nat. Microbiol..

[40] Altschul SF, Gish W, Miller W, Myers EW, Lipman DJ (1990). Basic local alignment search tool. J. Mol. Biol..

[41] Chen L-X, Anantharaman K, Shaiber A, Eren AM, Banfield JF (2020). Accurate and complete genomes from metagenomes. Genome Res..

[42] Peng Y, Leung HCM, Yiu SM, Chin FYL (2012). IDBA-UD: a de novo assembler for single-cell and metagenomic sequencing data with highly uneven depth. Bioinformatics.

[43] Li D, Liu C-M, Luo R, Sadakane K, Lam T-W (2015). MEGAHIT: an ultra-fast single-node solution for large and complex metagenomics assembly via succinct de Bruijn graph. Bioinformatics.

[44] Hyatt D (2010). Prodigal: prokaryotic gene recognition and translation initiation site identification. BMC Bioinforma..

[45] Edgar RC (2010). Search and clustering orders of magnitude faster than BLAST. Bioinformatics.

[46] Bushnell, B. BBMap: a fast, accurate, splice-aware aligner. https://www.osti.gov/biblio/1241166-bbmap-fast-accurate-splice-aware-aligner (2014).

[47] Liao Y, Smyth GK, Shi W (2014). featureCounts: an efficient general purpose program for assigning sequence reads to genomic features. Bioinformatics.

[48] Shen W, Le S, Li Y, Hu F (2016). SeqKit: a cross-platform and ultrafast toolkit for FASTA/Q file Manipulation. PLoS ONE.

[49] Li H (2009). The sequence alignment/Map format and SAMtools. Bioinformatics.

[50] Wick, R. R. Porechop: an adapter trimmer for Oxford Nanopore reads. https://github.com/rrwick/Porechop (2018).

[51] Kolmogorov M, Yuan J, Lin Y, Pevzner PA (2019). Assembly of long, error-prone reads using repeat graphs. Nat. Biotechnol..

[52] Katoh K, Misawa K, Kuma K-I, Miyata T (2002). MAFFT: a novel method for rapid multiple sequence alignment based on fast Fourier transform. Nucleic Acids Res..

[53] Capella-Gutiérrez S, Silla-Martínez JM, Gabaldón T (2009). trimAl: a tool for automated alignment trimming in large-scale phylogenetic analyses. Bioinformatics.

[54] Nguyen L-T, Schmidt HA, von Haeseler A, Minh BQ (2015). IQ-TREE: a fast and effective stochastic algorithm for estimating maximum-likelihood phylogenies. Mol. Biol. Evol..

[55] Letunic I, Bork P (2016). Interactive tree of life (iTOL) v3: an online tool for the display and annotation of phylogenetic and other trees. Nucleic Acids Res..

[56] Chan PP, Lin BY, Mak AJ, Lowe TM (2021). tRNAscan-SE 2.0: improved detection and functional classification of transfer RNA genes. Nucleic Acids Res..

[57] Jones P (2014). InterProScan 5: genome-scale protein function classification. Bioinformatics.

[58] Finn RD (2014). Pfam: the protein families database. Nucleic Acids Res..

[59] Aramaki T (2020). KofamKOALA: KEGG Ortholog assignment based on profile HMM and adaptive score threshold. Bioinformatics.

[60] Krogh A, Larsson B, von Heijne G, Sonnhammer EL (2001). Predicting transmembrane protein topology with a hidden Markov model: application to complete genomes. J. Mol. Biol..

[61] Yu NY (2010). PSORTb 3.0: improved protein subcellular localization prediction with refined localization subcategories and predictive capabilities for all prokaryotes. Bioinformatics.

[62] Nawrocki, E. P. *Structural RNA Homology Search and Alignment Using Covariance Models* (ProQuest Dissertations Publishing, 2009).

[63] Jumper J (2021). Highly accurate protein structure prediction with AlphaFold. Nature.

[64] Mirdita, M. et al. ColabFold - Making protein folding accessible to all. *Nature Methods*10.1038/s41592-022-01488-1 (2022). 10.1038/s41592-022-01488-1 PMC9184281 35637307

[65] DeLano, W. L. The PyMOL molecular graphics system. http://www.pymol.org (2002).

[66] Krissinel E, Henrick K (2004). Secondary-structure matching (SSM), a new tool for fast protein structure alignment in three dimensions. Acta Crystallogr. D. Biol. Crystallogr..

[67] Gilchrist, C. L. M. & Chooi, Y.-H. Clinker & clustermap.js: automatic generation of gene cluster comparison figures. *Bioinformatics*10.1093/bioinformatics/btab007 (2021). 10.1093/bioinformatics/btab007 33459763

[68] Hauser M, Steinegger M, Söding J (2016). MMseqs software suite for fast and deep clustering and searching of large protein sequence sets. Bioinformatics.

[69] Remmert M, Biegert A, Hauser A, Söding J (2011). HHblits: lightning-fast iterative protein sequence searching by HMM-HMM alignment. Nat. Methods.

[70] Söding J (2005). Protein homology detection by HMM-HMM comparison. Bioinformatics.

[71] Brown CT, Olm MR, Thomas BC, Banfield JF (2016). Measurement of bacterial replication rates in microbial communities. Nat. Biotechnol..

