# Supplementary material for: A widespread group of large plasmids in methanotrophic Methanoperedens archaea
Source: Nat Commun. 2022 Nov 18;13:7085. doi: 10.1038/s41467-022-34588-9 (PMC9674854; doi:10.1038/s41467-022-34588-9)
Supplement: Supplementary file 1 — Supplementary Information [file 41467_2022_34588_MOESM1_ESM.pdf]

## Supplementary Data for:

# A widespread group of large plasmids in methanotrophic *Methanoperedens* archaea

Marie C. Schoelmerich[1], Heleen T. Ouboter[2,5], Rohan Sachdeva[1], Petar Penev[1], Yuki Amano[3], Jacob West-Roberts[4], Cornelia U. Welte[2,5] and Jillian F. Banfield[1,4,6,7]*

**Affiliations:**

[1] Innovative Genomics Institute, University of California, Berkeley, CA, USA
[2] Department of Microbiology, Radboud University, Nijmegen, AJ, Netherlands
[3] Sector of Decommissioning and Radioactive Wastes Management, Japan Atomic Energy Agency, Ibaraki, Japan
[4] Environmental Science, Policy and Management, University of California, Berkeley, CA, USA
[5] Soehngen Institute of Anaerobic Microbiology, Radboud University, Nijmegen, AJ, Netherlands
[6] Earth and Planetary Science, University of California, Berkeley, CA, USA
[7] Lawrence Berkeley National Laboratory, Berkeley, CA, USA

* Corresponding author: jbanfield@berkeley.edu

**This PDF file includes:**

Supplementary figures S1-S9 and legends

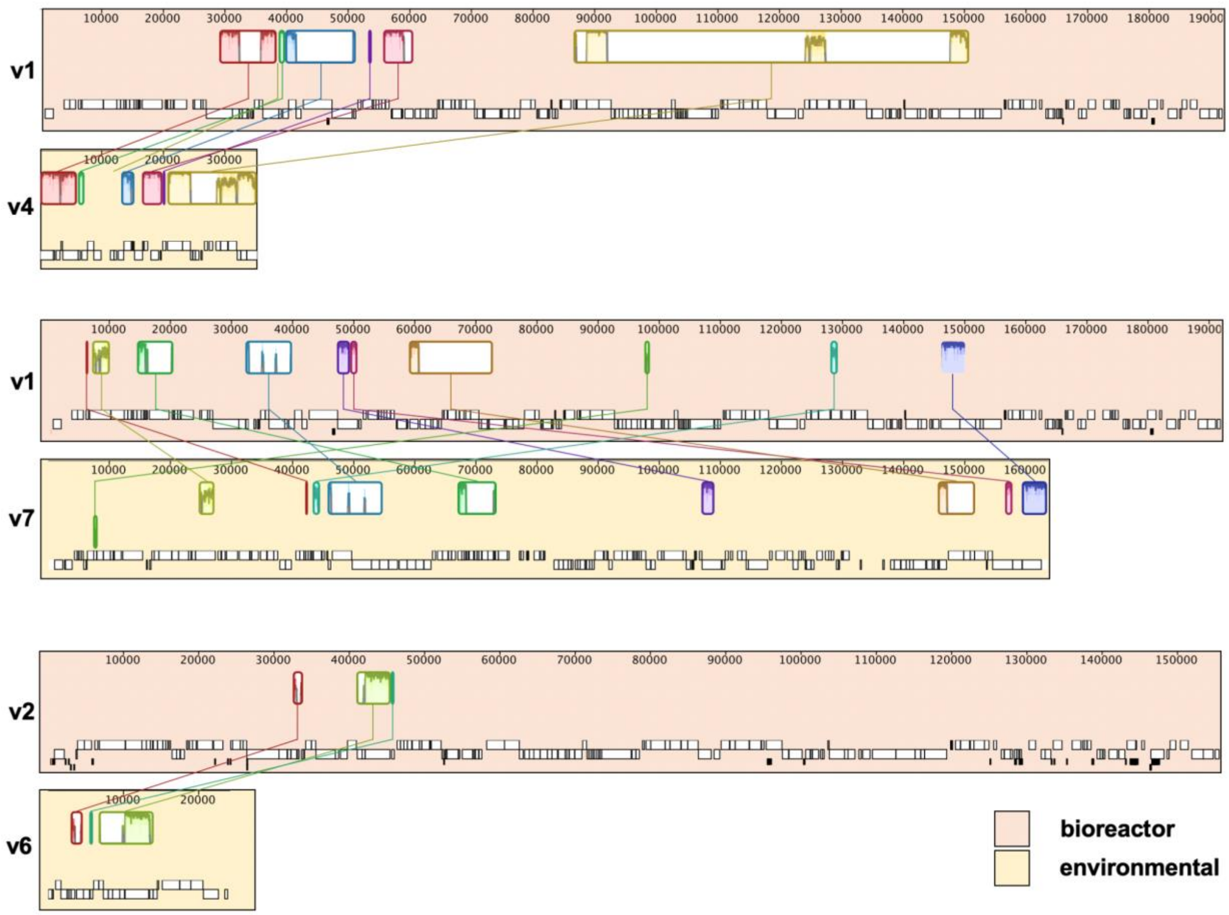


**Figure S1. Mauve genome alignments of plasmid fragments from environmental samples to the curated v1 or v2 plasmid genome.** Homologous regions of sequence shared by both plasmids (collinear blocks) are highlighted in the same color.

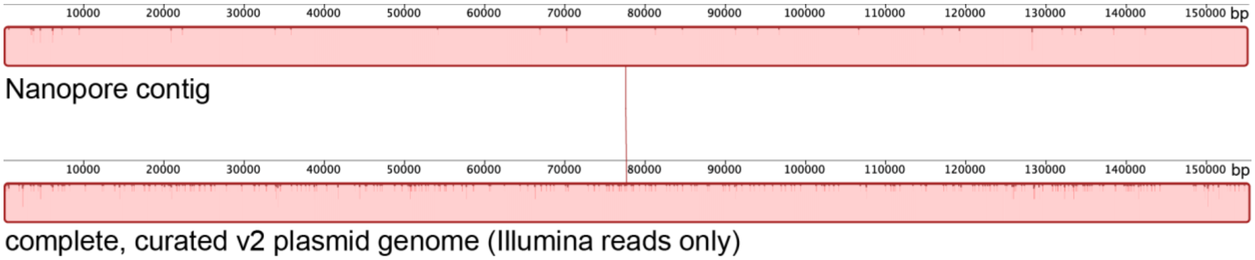


**Figure S2. Mauve genome alignment of a contig originating from an assembly of long reads (Nanopore) to the complete and curated v2 plasmid genome assembled from short reads (Illumina).** Homologous regions of sequence shared by both plasmids (collinear blocks) are highlighted in the same color. The single 153,309 bp Nanopore contig supports the complete genome throughout, with the exception of occasional single Nanopore basecall errors.

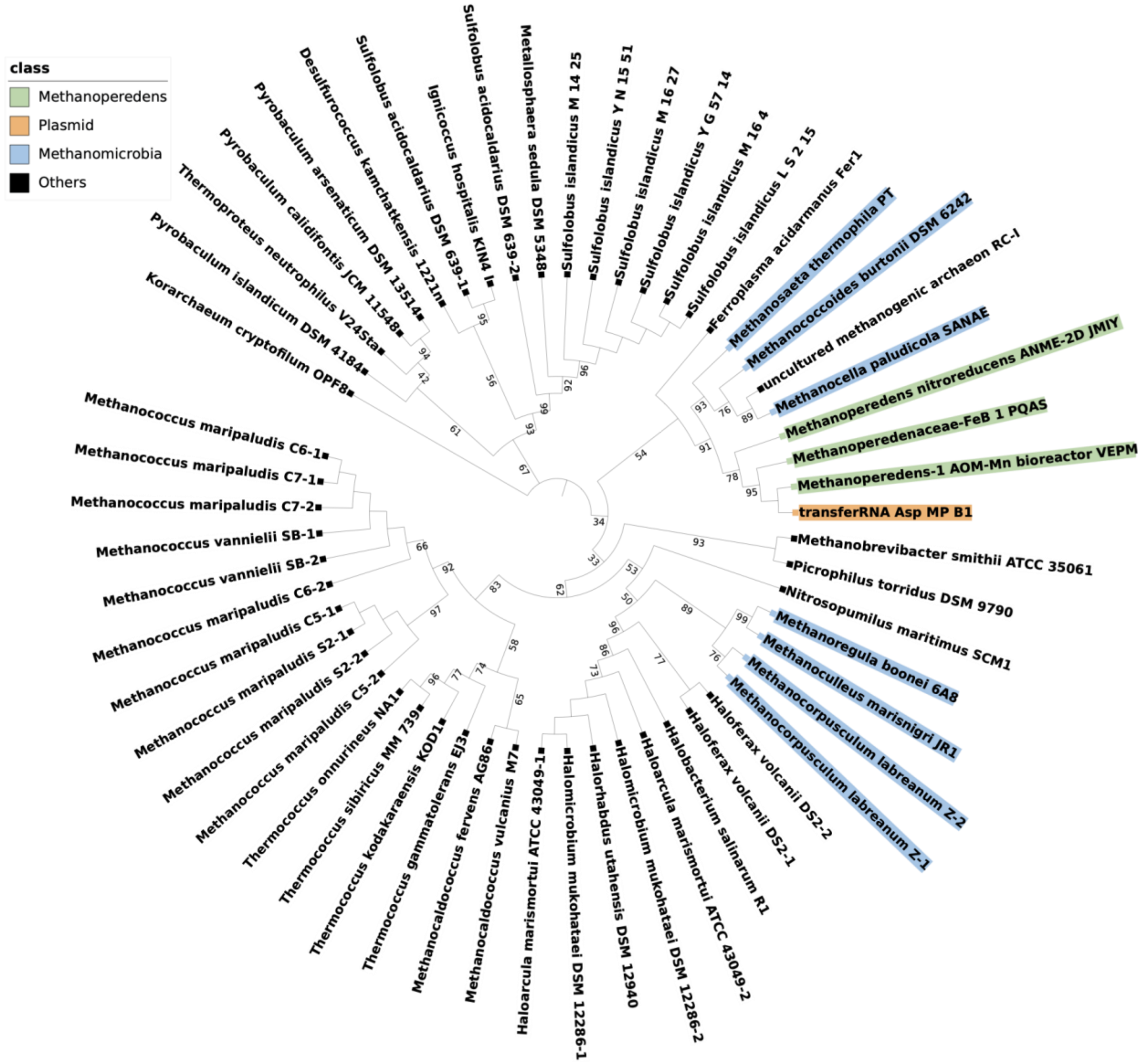


**Figure S3. Phylogenetic tree of archaeal Asp tRNAs.** Asp tRNA of HMp_v5 falls within a clade of other Asp tRNA from other *Methanoperedens* species but is absent from the host genome.

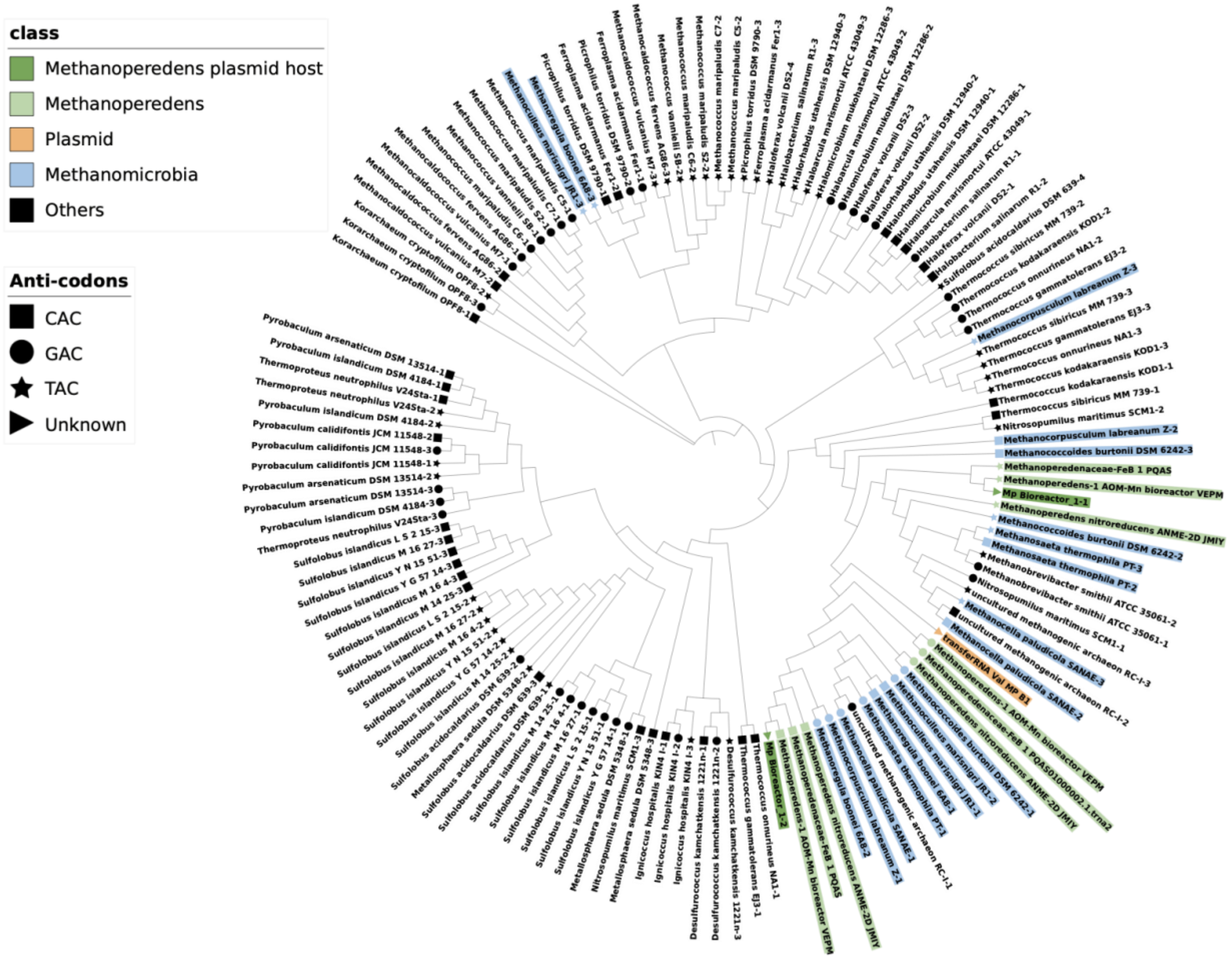


**Figure S4. Phylogenetic tree of archaeal Val tRNAs.** Val tRNA of HMp_v5 falls within a clade of other Val tRNA from *Methanomicrobia,* including the two from the host *Methanoperedens.*

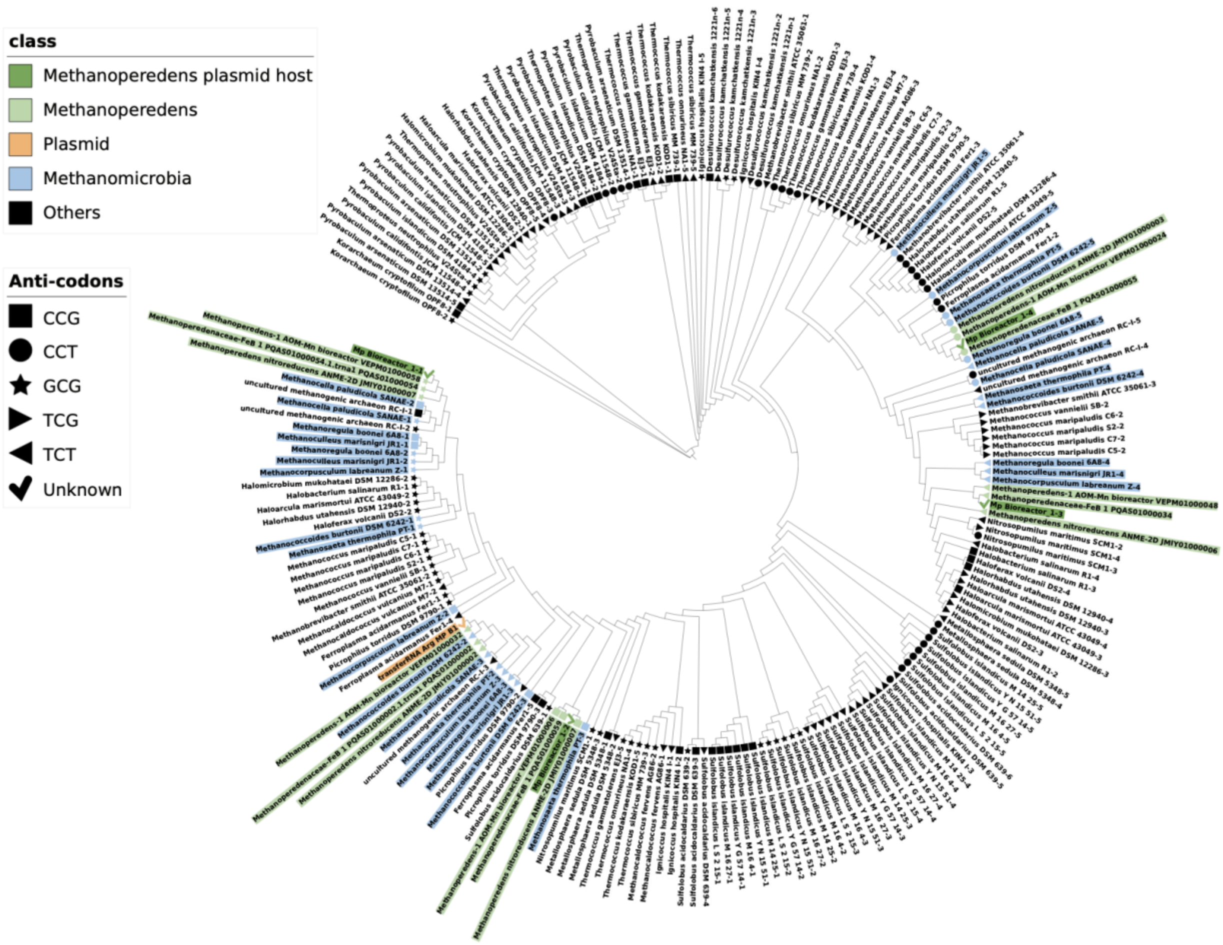


**Figure S5. Phylogenetic tree of archaeal Arg tRNAs.** Arg tRNA of HMp_v5 falls within a clade of other Asp tRNA from other *Methanoperedens* species*.* The host has four Arg tRNA, but with different anticodon types.

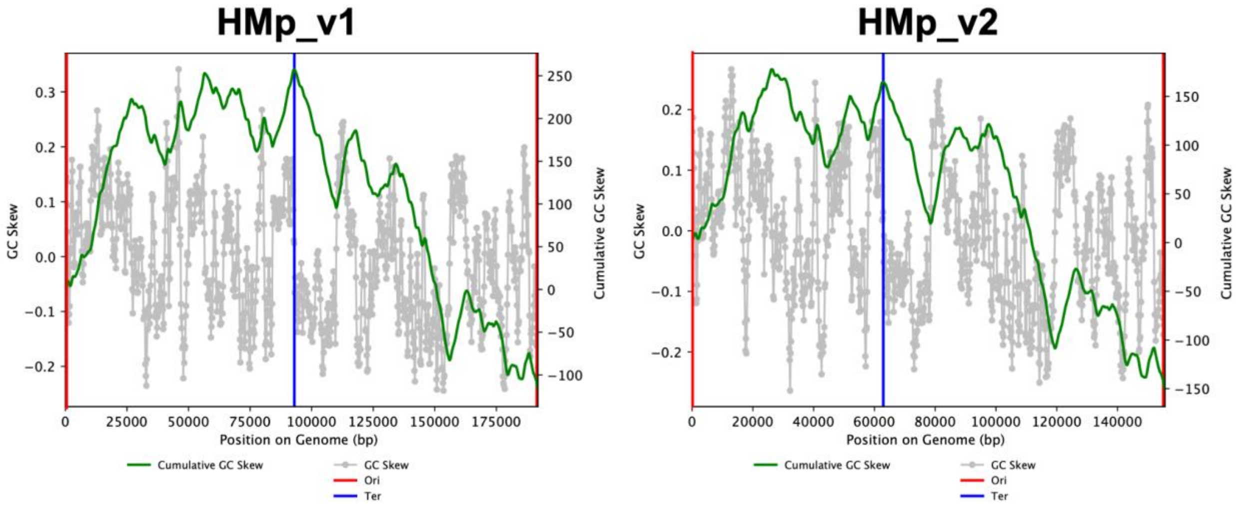


**Figure S6. GC skew analysis of HMp_v1 and HMp_v2.** Origin (Ori) of replication and termini (Ter) were calculated from cumulative GC skew.

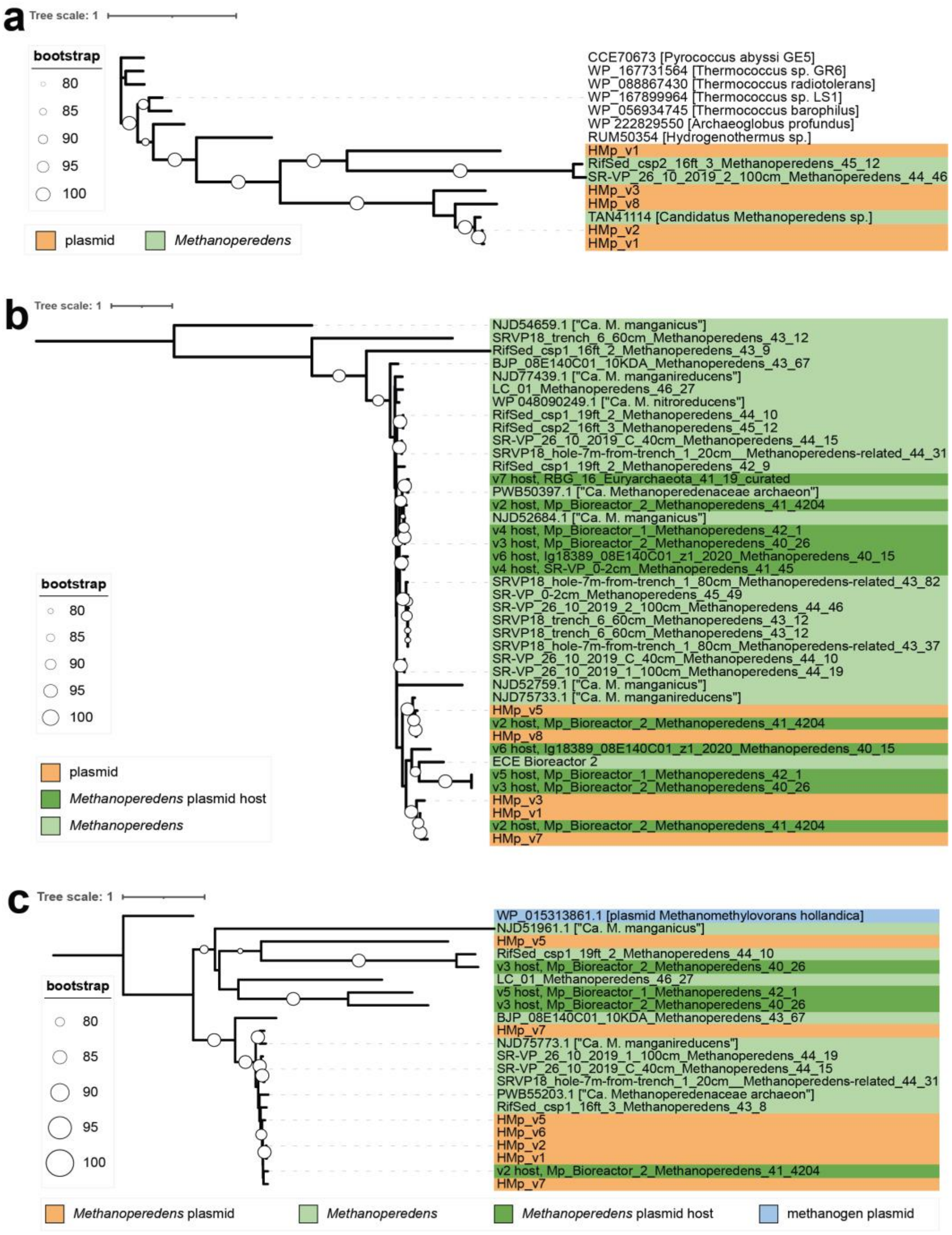


**Figure S7. Phylogenetic tree of plasmid enriched protein subfamilies comprising UvrD helicase, RadC and a helicase/nuclease. a.** Unrooted tree of UvrD helicase proteins (subfamily4932+selected top Blastp hits). Plasmid UvrD cluster together, are absent in the host genomes, but cluster with *Methanoperedens* genomes **b**. Phylogenetic tree of RadC proteins (subfamily7324) rooted on NJD54659.1 from "*Ca.* M. manganicus". Plasmid proteins cluster together with a group of homologues from plasmid host *Methanoperedens* and an unclassified extrachromosomal element from Bioreactor 2. **c.** Phylogenetic tree of helicase/nuclease protein (subfamily3527) rooted on protein from *M. hollandica* plasmid. The plasmid proteins cluster together. The second versions on v5 and v7 are more related to proteins found on *Methanoperedens* genomes.

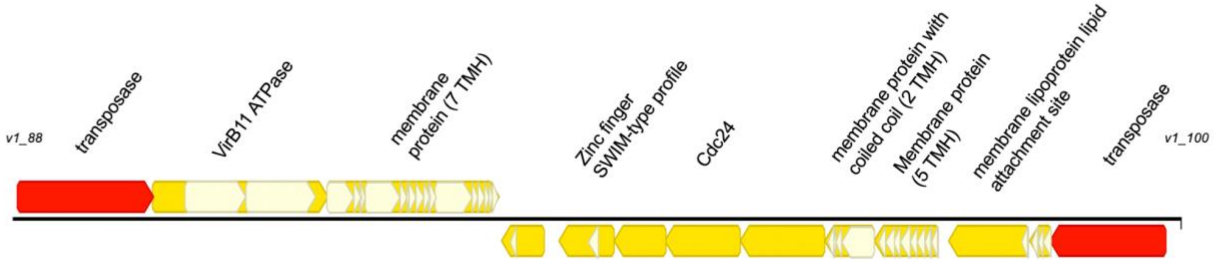


**Figure S8. Two gene clusters on HMp_v1 encoding putative membrane complexes.**

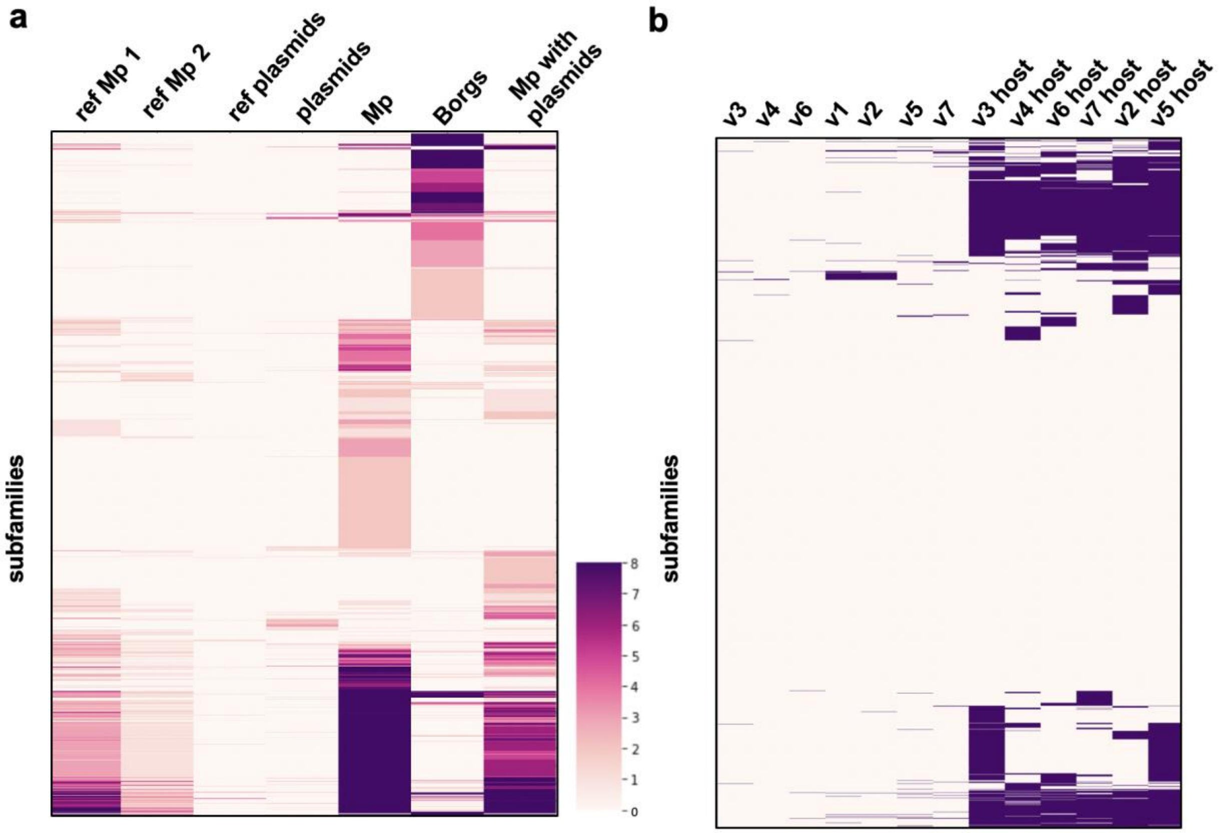


**Figure S9. Protein subfamilies of plasmids, *Methanoperedens* with or without plasmids, Borgs, reference *Methanoperedens* and reference plasmids. A.** Heatmap showing protein subfamilies (subfamilies ≥ 8 are all shown in dark purple). Reference *Methanoperedens* group 1 (ref Mp 1) comprise proteomes of "*Ca*. M. nitroreducens", "*Ca*. M. ferrireducens", "*Ca*. manganicus", ref Mp 2 is proteome of "*Ca*. M. manganireducens", reference plasmids comprise 8 plasmid proteomes of methanogens. **B.** Presence-absence map of plasmid proteomes (v1-v7) and their inferred *Methanoperedens* host's proteomes.
