# Supplementary material for: A widespread group of large plasmids in methanotrophic Methanoperedens archaea
Source: Nat Commun. 2022 Nov 18;13:7085. doi: 10.1038/s41467-022-34588-9 (PMC9674854; doi:10.1038/s41467-022-34588-9)
Supplement: Supplementary file 2 — Description of Additional Supplementary Files [file 41467_2022_34588_MOESM2_ESM.pdf]

Description of Additional Supplementary Files:

Supplementary Data 1: plasmids v1 v2

Supplementary Data 2: metaT plasmids

Supplementary Data 3: all protein subfamilies

Supplementary Data 4: protein dataset

Supplementary Data 5: plasmid v2 update

Supplementary Data 6: Nanopore contig
