# Supplementary material for: A widespread group of large plasmids in methanotrophic Methanoperedens archaea
Source: Nat Commun. 2022 Nov 18;13:7085. doi: 10.1038/s41467-022-34588-9 (PMC9674854; doi:10.1038/s41467-022-34588-9)
Supplement: Supplementary file 5 — Reporting Summary [file 41467_2022_34588_MOESM5_ESM.pdf]

nature portfolio

Corresponding author(s): Jill Banfield

Last updated by author(s): Oct 22. 2022

Reporting Summary

Nature Portfolio wishes to improve the reproducibility of the work that we publish. This form provides structure for consistency and transparency in reporting. For further information on Nature Portfolio policies, see our Editorial Policies and the Editorial Policy Checklist.

Statistics

For all statistical analyses, confirm that the following items are present in the figure legend, table legend, main text, or Methods section.

n/a	Confirmed	
☐	☒	The exact sample size (*n*) for each experimental group/condition, given as a discrete number and unit of measurement
☐	☒	A statement on whether measurements were taken from distinct samples or whether the same sample was measured repeatedly
☐	☒	The statistical test(s) used AND whether they are one- or two-sided<br>*Only common tests should be described solely by name; describe more complex techniques in the Methods section.*
☐	☒	A description of all covariates tested
☐	☒	A description of any assumptions or corrections, such as tests of normality and adjustment for multiple comparisons
☐	☒	A full description of the statistical parameters including central tendency (e.g. means) or other basic estimates (e.g. regression coefficient) AND variation (e.g. standard deviation) or associated estimates of uncertainty (e.g. confidence intervals)
☐	☒	For null hypothesis testing, the test statistic (e.g. *F*, *t*, *r*) with confidence intervals, effect sizes, degrees of freedom and *P* value noted<br>*Give P values as exact values whenever suitable.*
☒	☐	For Bayesian analysis, information on the choice of priors and Markov chain Monte Carlo settings
☐	☒	For hierarchical and complex designs, identification of the appropriate level for tests and full reporting of outcomes
☒	☐	Estimates of effect sizes (e.g. Cohen's *d*, Pearson's *r*), indicating how they were calculated

Our web collection on statistics for biologists contains articles on many of the points above.

Software and code

Policy information about availability of computer code

Data collection	Geneious Prime (v. 2021.2.2)<br>BBTools (v38.79)<br>IDBA-UD (v1.1.3)<br>Megahit (v1.2.9)<br>Sickle (v1.33)
Data analysis	Python (v. 3.8.3)<br>progressive Mauve algorithm (v. 2.3.1)<br>Prodigal (v2.6.3)<br>USEARCH (v10.0.240)<br>featureCounts (v2.0.3)<br>SeqKit (v0.12.0)<br>MAFFT (v7.453)<br>trimal (v1.4.rev15),<br>IQ-TREE (v1.6.12),<br>GtRNAdb (release 19),<br>iTOL (v.6.5.7),

InterProScan (v5.51-85.0),
HMMER (v3.3),
TMHMM (v2.0),
tRNAscan (v.2.0.9),
SSU-ALIGN (v0.1.1),
AlphaFold2,
LocalColabFold (v1.2.0),
PyMOL (v2.3.4),
PDBeFold (v2.58),
clinker (v0.0.21),
MMseqs2 (a62ea9a9952aab45a24725c1208440bed66e8ec6),
HHblits (v3.2.0),
HHsearch (v.3.2.0),
SAMtools (v1.12).
GC skew analyses were performed using a python code available on github (https://github.com/christophertbrown/iRep/blob/master/iRep/gc_skew.py).

For manuscripts utilizing custom algorithms or software that are central to the research but not yet described in published literature, software must be made available to editors and reviewers. We strongly encourage code deposition in a community repository (e.g. GitHub). See the Nature Portfolio guidelines for submitting code & software for further information.

Data

Policy information about availability of data

All manuscripts must include a data availability statement. This statement should provide the following information, where applicable:

- Accession codes, unique identifiers, or web links for publicly available datasets
- A description of any restrictions on data availability
- For clinical datasets or third party data, please ensure that the statement adheres to our policy

Metagenomics and metatranscriptomics sequencing reads, and newly released plasmid genomes and "Candidatus Methanoperedens spp." metagenomes reported in this paper are available under NCBI BioProject: PRJNA850006. Reference datasets comprising additional "Candidatus Methanoperedens spp." and Borg genomes are available under NCBI BioProject: PRJNA866293. The HMp_v2 contig assembled from Nanopore long-reads is provided in Supplementary Data 6. Sequence databases used include KEGG, UniRef100, UniProt, pfam, ggkbase (ggkbase.berkeley.edu).

Human research participants

Policy information about studies involving human research participants and Sex and Gender in Research.

Reporting on sex and gender	not applicable.
Population characteristics	not applicable.
Recruitment	not applicable.
Ethics oversight	not applicable.

Note that full information on the approval of the study protocol must also be provided in the manuscript.

Field-specific reporting

Please select the one below that is the best fit for your research. If you are not sure, read the appropriate sections before making your selection.

[x] Life sciences   [ ] Behavioural & social sciences   [ ] Ecological, evolutionary & environmental sciences

For a reference copy of the document with all sections, see nature.com/documents/nr-reporting-summary-flat.pdf

Life sciences study design

All studies must disclose on these points even when the disclosure is negative.

Sample size	There were no statistical methods to determine sample sizes. The total samples size used for the metagenomics experiment included 2 bioreactors and 9 environmental sites. The bioreactors were sampled twice for metagenomic datasets and in triplicate for metatranscriptomic datasets. The attempts to isolate the plasmids were performed in duplicate for each bioreactor. No significant enrichment was achieved, however one sample resulted in a better assembly of plasmid version HMp-v5 and was thus used in the final dataset.

Data exclusions	Sequencing reads with low quality scores were excluded, as is commonly performed prior to assembly of short read data. Metagenomic datasets from the attempts to isolate the plasmids were also excluded, as they did not result in improved genome sequences, with one exception being the genome MP_B1_plasmid_isolate_R2_Ooijpolder_MG_HMp-v5_40_44.
Replication	Metagenomic datasets from the bioreactors were successfully constructed using technical duplicates. Metatranscriptomic datasets from the bioreactors were constructed using technical triplicates. Metagenomic datasets from the plasmid isolation attempts were constructed using technical duplicates.<br>> Replication of Cultivation Conditions<br>The bioreactor cultures have been growing for ~7 years. This time frame thus did not allow for making biological replicates of the bioreactors.<br>> Replication of Environmental Samples<br>The genomes published herein were discovered in our own (unpublished) ggkbase database. The data originate from different environmental sampling campaigns and were thus not subject to replication.
Randomization	>Samples<br>Samples from each bioreactor formed one experimental subgroup (bioreactor 1 and bioreactor 2 make up the bioreactor group). The samples originating from different environdmental sites where plasmids (and one extrachromosomal element [ECE]) and Methanoperedens were discovered correspond to the environmental experimental group.<br>> Enrichment analyses from protein family clustering<br>Protein family clustering of 39,410 proteins (Table S4) and subsequent enrichment analyses were performed to evaluate which proteins are enriched or exclusively found in plasmids of Methanoperedens. The enrichment analyses were performed using the Fisher exact statistic using the fisher_exact funtion in scipy.stats (alternative='two-sided') was used. .
Blinding	Blinding was not performed because it was not applicable to this study. The study required the investigators to know the true groupings of the data to understand the results at the onset of the analysis.

Reporting for specific materials, systems and methods

We require information from authors about some types of materials, experimental systems and methods used in many studies. Here, indicate whether each material, system or method listed is relevant to your study. If you are not sure if a list item applies to your research, read the appropriate section before selecting a response.

Materials & experimental systems

n/a	Involved in the study
☒	☐ Antibodies
☒	☐ Eukaryotic cell lines
☒	☐ Palaeontology and archaeology
☐	☒ Animals and other organisms
☒	☐ Clinical data
☒	☐ Dual use research of concern

Methods

n/a	Involved in the study
☒	☐ ChIP-seq
☒	☐ Flow cytometry
☒	☐ MRI-based neuroimaging

Animals and other research organisms

Policy information about studies involving animals; ARRIVE guidelines recommended for reporting animal research, and Sex and Gender in Research

Laboratory animals	not applicable.
Wild animals	not applicable.
Reporting on sex	not applicable.
Field-collected samples	Samples were collected from a wetland (Lake County, CA, USA), discharge from an abandoned mine (Corona Mine in Lake County, CA, USA), sediment from an aquifer (Rifle, CO, USA) and a borehole (Horonobe, JP).
Ethics oversight	*Identify the organization(s) that approved or provided guidance on the study protocol, OR state that no ethical approval or guidance was required and explain why not.*

Note that full information on the approval of the study protocol must also be provided in the manuscript.
